# Interaction between dietary branched-chain amino acids and genetic risk score on the risk of type 2 diabetes in Chinese

**DOI:** 10.1186/s12263-021-00684-6

**Published:** 2021-03-04

**Authors:** Weiqi Wang, Haiyang Jiang, Ziwei Zhang, Wei Duan, Tianshu Han, Changhao Sun

**Affiliations:** National Key Discipline, Department of Nutrition and Food Hygiene, School of Public Health, Harbin Medical University, 157 Baojian Road, Harbin, 150081 People’s Republic of China

**Keywords:** Branched-chain amino acid, Genetic risk score, Interaction, Type 2 diabetes, Chinese adult

## Abstract

**Background and objectives:**

Previous studies have found the important gene-diet interactions on type 2 diabetes (T2D) incident but have not followed branched-chain amino acids (BCAAs), even though they have shown heterogeneous effectiveness in diabetes-related factors. So in this study, we aim to investigate whether dietary BCAAs interact with the genetic predisposition in relation to T2D risk and fasting glucose in Chinese adults.

**Methods:**

In a case-control study nested in the Harbin Cohort Study on Diet, Nutrition and Chronic Non-Communicable Diseases, we obtained data for 434 incident T2D cases and 434 controls matched by age and sex. An unweighted genetic risk score (GRS) was calculated for 25 T2D-related single nucleotide polymorphisms by summation of the number of risk alleles for T2D. Multivariate logistic regression models and general linear regression models were used to assess the interaction between dietary BCAAs and GRS on T2D risk and fasting glucose.

**Results:**

Significant interactions were found between GRS and dietary BCAAs on T2D risk and fasting glucose (*p* for interaction = 0.001 and 0.004, respectively). Comparing with low GRS, the odds ratio of T2D in high GRS were 2.98 (95% CI 1.54–5.76) among those with the highest tertile of total BCAA intake but were non-significant among those with the lowest intake, corresponding to 0.39 (0.12) mmol/L versus − 0.07 (0.10) mmol/L fasting glucose elevation per tertile. Viewed differently, comparing extreme tertiles of dietary BCAAs, the odds ratio (95% CIs) of T2D risk were 0.46 (0.22–0.95), 2.22 (1.15–4.31), and 2.90 (1.54–5.47) (fasting glucose elevation per tertile: − 0.23 (0.10), 0.18 (0.10), and 0.26 (0.13) mmol/L) among participants with low, intermediate, and high genetic risk, respectively.

**Conclusions:**

This study indicated that dietary BCAAs could amplify the genetic association with T2D risk and fasting glucose. Moreover, higher BCAA intake showed positive association with T2D when genetic predisposition was also high but changed to negative when genetic predisposition was low.

**Supplementary Information:**

The online version contains supplementary material available at 10.1186/s12263-021-00684-6.

## Introduction

According to the latest reports from International Diabetes Federation, 451 million adults worldwide were living with diabetes, a vast majority of whom had type 2 diabetes (T2D) [1, 2]. Compelling evidence has shown that the etiology of T2D includes environmental factors, like diet, smoking, and physical activity [3]. Furthermore, a genetic component also exists for the disease; the concordance for T2D in monozygotic twins has been found stronger than in dizygotic twins [4], and family history shows an independent prediction for T2D [5].

With the huge development in genome-wide association study (GWAS) in the past decade, the lead single nucleotide polymorphisms (SNP) associated with T2D-related traits have been identified and replicated in many regions, which can be used to characterize individual genetic predisposition to T2D [6]. On the basis of scientific evidence, many susceptibility genes of T2D have been identified to interact with environmental factors like diet [7–9]. Investigation of gene–environment interactions are necessary to further explore the underlying pathophysiology of T2D and could potentially be used to improve disease risk stratification and providing individual lifestyle recommendations corresponding to the risk stratification [10].

Branched-chain amino acids (BCAAs, including leucine, isoleucine, and valine) are essential amino acids that need to be obtained from the diet and comprise almost 50% of the essential amino acids in food [11], mainly coming from meat, fish, milk, and eggs. BCAAs have a critical role in protein synthesis, insulin sensitivity, and glucose homeostasis [12, 13]. An overwhelming number of publications have consistently demonstrated that circulating BCAAs can credibly predict the development of insulin resistance and T2D risk [14–16], independent of typical risk factors like sex, age, and BMI [17]. Circulating BCAAs and toxic metabolites can impair mitochondrial oxidation of glucose, leading to mitochondrial stress and impaired insulin excretion and action [15]. Meanwhile, in T2D and IFG patients, damaged mitochondrial function may reduce the capacity of the mitochondria to catabolize BCAAs, resulting in elevated levels of BCAAs and related metabolite in a vicious cycle [18]. Dietary BCAAs influenced plasma level along with individual metabolism ability [19] and showed conflicting relationships with T2D. In prospective cohorts of the Nurses’ Health Study (NHS), NHS II, the Health Professionals Follow-up Study, and the Women’s Health Initiative study, dietary BCAAs were positively associated with T2D risk [20, 21]. But in the cohort of Takayama study [22] and in numerous rodent models [23], increasing dietary BCAAs decreased T2D risk and improved T2D-related parameters, such as insulin sensitivity and glycaemia levels [24]. However, no research has ever focused on the combining effect of dietary BCAAs and genetic susceptibility to T2D.

So in this article, we sought to explore whether the interaction between dietary BCAAs and the genetic susceptibility evaluated by a genetic risk score (GRS) on T2D risk and fasting glucose exists in a prospective nested case-control study from a Chinese adults cohort.

## Methods

### Study population

The Harbin Cohort Study on Diet, Nutrition and Chronic Non-Communicable Diseases is a prospective cohort study consisted of 9734 Chinese adults at study initiation in 2012. Information about dietary habits, demographic characteristics, physical condition, and lifestyle was administered by trained healthcare staff using a structured questionnaire. Anthropometric characteristics were collected at the same time. The details have been previously published in the previous study [25]. After excluding those who had T2D, deficient dietary data and/or blood samples, implausible energy intake values (< 800 kcal or > 4500 kcal for males; < 500 kcal or > 4000 kcal for females) at the baseline survey and those who lack anthropometric measures in follow-up, 4964 participants remained in the cohort, including 434 new diagnosed diabetes cases. The cases were matched to 434 non-diabetic control subjects on age and sex, because age and sex were confounders in the whole cohort that were related to both independent variables and dependent variables.

This cohort study protocol was approved by the Ethics Committee of the Harbin Medical University and was administrated in conformity to the Declaration of Helsinki. All participants offered written contents.

### Questionnaire survey

Long-term habitual dietary intakes were estimated according to food frequency questionnaire (FFQ) by trained dietary interviewers at baseline visit, including 103 food items from 14 food groups which are rice, wheaten food, beans and its products, potato starch and its products, fruits, vegetables, poultry and its products, livestock and its products, fish and its products, milk and its products, eggs and its products, beverage, ice cream, and snack. The validity and reproducibility of the FFQ was manifested by good correlation of food intake with that measured by a 3-day dietary record with the correlation coefficients to be 0.61–0.70. We used the China Food Composition Tables to calculate intakes of isoleucine (in mg/day), leucine (in mg/day), valine (in mg/day), total BCAAs (in mg/day), and protein (in g/day) (Supplementary Table 1).

Lifestyles and physical data were collected meanwhile. Exercise regularity was defined as recreational or sport physical activity performed at least 30 min for three or more days per week. Current smokers were defined as those who have smoked at least 100 cigarettes lifetime and smoke every day or some days now. Current drinkers were defined as those who consumed ≥ 1 alcoholic drink each month in the past 12 months before the survey. Family history of diabetes was defined as those whose first-degree relatives suffered from diabetes. The presence of cardiovascular disease at baseline was identified by self-report of a history of cardiovascular disease diagnosis in a structured questionnaire.

### Anthropometric measurements and circulating BCAA measurement

Anthropometric indices were measured by trained medical workers according to a standard protocol. Weight, height, and waist circumference were measured in light clothing and without shoes to the nearest 0.1 kg and 0.1 cm, respectively. BMI was calculated as weight divided by the square of the height in meters. Blood pressures were measured 3 times on the right arm of each participant after a 10-min rest, and the mean values were used for analysis.

Circulating BCAAs were analyzed by ultrahigh performance liquid chromatography–triple quadrupole mass spectrometry. UPLC–TQ–MS analysis was performed using a Waters ACQUITY UPLC system (Waters Corporation, Milford, MA, USA) coupled to a Waters Xevo TQD Mass Spectrometer (Waters Corporation, Manchester, UK) and a 2-ml aliquot of the sample solution was injected into an ACQUITY UPLCTM HILIC column (Waters Corporation, Milford, MA, USA) [26].

### Biochemical analyses and outcome measures

An oral glucose tolerance test (OGTT) was carried out for each cohort participant except those self-reporting diabetes according to the World Health Organization guidelines. Fasting and postprandial blood samples (fasting over 10 h and 2 h after drinking a 75-g glucose-containing water) were collected for biochemical assessment. After collection, plasma samples were kept in a portable, insulated bag with ice packs (at about 0–4 °C) and were processed within 6 h for long-term storage at − 80 °C. Fasting blood glucose and 2-h glucose was measured quantitatively with an auto-analyzer (Hitachi 7100 Auto-analyzer, Tokyo, Japan).

Based on the OGTT, T2D cases were defined as fasting blood glucose ≥ 7.0 mmol/L and/or 2-h glucose ≥ 11.1 mmol/L and/or HbA1c ≥ 40 mmol/mol (6.5%) or someone who self-report T2D and take control measures.

### DNA extraction and genotype determination

Peripheral venous blood samples were collected from each participant. Genomic DNA was extracted from peripheral blood leukocytes on a Tecan Freedom EVO platform (Tecan, Switzerland) using the Mag-Bind Blood DNA Kit (CWBIO, China)**.** Single nucleotide polymorphisms (SNPs) genotyping work was performed using a custom-by-design 48-Plex SNPscan^TM^ Kit (Cat#:G0104; Genesky Biotechnologies Inc., Shanghai, China). SNPs were selected from their consistent associations with impaired insulin synthesis, secretion, action, or other T2D-related traits in genome-wide association studies and candidate gene studies, especially in Chinese or Asian, and had to meet the following criteria: fitting Hardy-Weinberg equilibrium, genotyping success rates exceed 95%, and the genotyping concordance of replicated quality-control samples exceed 95%. On these basics, 25 SNP were incorporated in our study [27–41]. The details of these SNPs are shown in Supplementary Table 2.

### Genetic risk score calculation

To evaluate the combined effects of the 25 SNPs on diabetes risk and avoid the potential over-fitting [42], an additive genetic model was used to construct unweighted GRS, which assumed that each SNP acts independently on the risk of T2D. Unweighted GRS was calculated for each participant by summation of the number of risk alleles (0, 1, 2) across the 25 variants. For individuals with missing values of SNP, GRSs were standardized to those with complete data by the following equation: GRS = (total number risk alleles/number of non-missing genotypes × 2) × 50, supposing that the missing genotypes were not associated to disease status [43].

### Statistical analyses

BCAAs intakes were highly correlated with total protein intake (total BCAAs: *r* = 0.953, *P* < 0.0001) in partial correlation analysis. Therefore, nutrient densities were used to reduce the likelihood of multicollinearity by which the BCAAs intakes were characterized as a proportion of total protein intake [44]. Dietary BCAAs, circulating BCAAs, and GRS were shown in both continuous and classified mode, which were classified into 3 groups.

Odds ratios (ORs) and 95% CIs were calculated using the logistic regression model. The matching pairs by sex and age were broken in subgroup stratified analyses, so using conditional logistic regression model on matching factor may cause data loss. On this basis, multivariable unconditional logistic models were used to assess the main associations of the GRS and dietary BCAAs with T2D risk, the associations between each additional one risk allele and change in T2D risk according to dietary BCAAs in thirds, and the associations between each 10 mg/g protein increase in dietary BCAAs and T2D risk according to genetic risk subgroups [45]. We examined the interactions between dietary BCAAs and GRS by including the main effects as well as the corresponding interaction terms in the models in the same time (for example, dietary isoleucine × GRS). For the outcome of fasting glucose, we changed the model into multivariable linear regression models to calculate the effect size on fasting glucose as well as generalized linear models to measure the difference among groups and analyzed in the same way. In addition, these models were also applied to research the relation of GRS to circulating BCAAs. Potential confounders were adjusted in the multivariable analyses, including age (continuous), sex (male/female), body mass index (continuous), physical activity (mild, moderate, severe), drinking (never, past, or current), smoking (never, past, or current), family history of diabetes (yes/no), and total energy intakes (continuous). Analyzing the relationship between GRS and circulating BCAAs further adjusted for corresponding dietary BCAAs. When the missing data for covariates were fewer than 5%, we replaced the missing values by the median values.

In this nested case-control study, we measured the differences in baseline characteristics by general linear model for continuous variables and chi-square test for dichotomous variables. We used SPSS software, version 25.0, for statistical analyses and *p* < 0.05 was considered statistically significant, all tests were 2-tailed. Multiple testing correction was not applied given that we had examined only one genetic instrument (GRS) and one outcome in per model.

## Results

### Baseline characteristics of study population

A total of 434 incident cases were identified during follow-up for Harbin Cohort Study. The characteristics of participants at baseline in the nested case-control study are presented in Table 1. Participants with T2D had higher BMI, waist circumferences, circulating BCAAs, and higher prevalence of hypertension and hyperlipid compared with control.

**Table 1:** Baseline characteristics of study variables in T2D case and non-diabetic control subjects

	Non-diabetic control (*n* = 434)	T2D case (*n* = 434)	*P* value*
Age (years)	51.4 (0.4)	52.1 (0.4)	0.25
Male [*n*, (%)]	167 (38.5)	167 (38.5)	1.00
BMI (kg/m2)	24.5 (0.2)	26.3(0.2)	< 0.001
Waist circumferences (cm)	85.0 (0.5)	90.1 (0.5)	< 0.001
Current smokers [*n*, (%)]	76 (17.5)	79 (18.2)	0.87
Current drinkers [*n*, (%)]	171 (39.4)	160 (36.9)	0.49
Exercised regularly [*n*, (%)]	212 (48.8)	195 (44.9)	0.28
Energy intake (kcal/day)	2357 (41.7)	2409 (41.5)	0.38
Family history of T2D [*n*, (%)]	56 (12.9)	81 (18.7)	0.025
Hypertension [*n*, (%)]	83 (19.2)	128 (29.7)	< 0.001
Hyperlipid [*n*, (%)]	96 (22.2)	125 (29.3)	0.019
Coronary [*n*, (%)]	87 (20.0)	95 (21.9)	0.56
Dietary BCAAs (mg/g protein)			
Total BCAAs	207(1.9)	211 (1.9)	0.12
Isoleucine	51.6 (0.4)	52.5 (0.4)	0.12
Leucine	97.0 (1.0)	99.2 (1.0)	0.11
Valine	58.3 (0.5)	59.3 (0.5)	0.15
Circulating BCAAs (umol/L)			
Total BCAAs	140 (25.2)	147 (31.0)	0.014
Isoleucine	23.1 (7.9)	25.5 (10.6)	< 0.001
Leucine	14.7 (5.8)	17.0 (9.4)	< 0.001
Valine	97.4 (23.4)	106 (20.9)	< 0.001
Protein(g/day)	62.4 (1.8)	63.0 (1.8)	0.80
Fat(g/day)	74.4 (1.5)	76.8 (1.5)	0.25
Carbohydrate(g/day)	364 (7.2)	369 (7.2)	0.58
Fast glucose (mmol/l)	4.4 (0.09)	6.7(0.09)	< 0.001

Data represent the mean ± SD or number (%)

*BCAAs* branched-chain amino acids, *BMI* body mass index, *T2D* type 2 diabetes

*P values were calculated by using the general linear model for continuous variables and chi-square tests for categorical variables.

### Genetic association with T2D risk and fasting glucose according to dietary BCAAs in thirds

The GRS calculated from 25 T2D-related SNPs by unweighted additive model ranged from 12 to 33(median: 22) and then divided equally into three groups: low (< 22), moderate (22–24), and high (≥ 25). In general, the GRS was positively associated with T2D incident and fasting glucose (*p* for trend = 0.007 and < 0.001, respectively). Dietary BCAAs showed similar associations with T2D risk but not fasting glucose (Supplementary Table 3).

Comparing with low GRS, the odds ratio of T2D in high GRS was more prominent among participants with the highest tertile of total BCAAs intake (OR 2.98; 95% CI 1.54, 5.76) than those with the lowest total BCAA intakes (OR 0.63; 95% CI 0.32, 1.24) (*p* for interaction = 0.001 for total BCAAs) (Table 2). The genetic associations with T2D risk were still significantly enhanced with high intakes of BCAAs when GRS was shown in a continuous form (*p* for interaction < 0.001 for total BCAAs) (Table 2). The odds ratio of T2D risk per 1 risk allele increment were 1.13 (95% CI 1.06, 1.21), 1.10 (1.02–1.17), and 0.95 (95% CI 0.88, 1.01) among those in the high, medium, and low tertile of total BCAAs. When viewed together, the genetic associations with T2D risk enhanced in participants who intake high total and individual BCAAs.

**Table 2:** The odds ratio of T2D by GRS in classified and continuous form according to dietary BCAAs in thirds^a^

Dietary BCAAs (mg/g protein)	GRS in classified mode					GRS in continuous mode		
	< 22	22–24	≥ 25	*P* for Trend	*P* for interaction	Per 1 allele	*P* value	*P* for interaction
Total BCAAs					0.001			< 0.001
T1 (*n* = 289)	1	0.71 (0.34–1.36)	0.63 (0.32–1.24)	0.19		0.95 (0.88–1.01)	0.11	
T2 (*n* = 290)	1	1.00 (0.53–1.90)	2.22 (1.12–4.12)	0.012		1.10 (1.02–1.17)	0.006	
T3 (*n* = 289)	1	2.30 (1.19–4.46)	2.98 (1.54–5.76)	0.001		1.13 (1.06–1.21)	< 0.001	
Isoleucine					< 0.001			< 0.001
T1 (*n* = 289)	1	0.63 (0.32–1.22)	0.58 (0.29–1.13)	0.12		0.94 (0.88–1.01)	0.08	
T2 (*n* = 290)	1	1.15 (0.61–2.18)	2.39 (1.29–4.44)	0.006		1.10 (1.03–1.17)	0.005	
T3 (*n* = 289)	1	2.13 (1.11–4.07)	2.95 (1.53–5.68)	0.001		1.13 (1.06–1.21)	< 0.001	
Leucine					0.001			< 0.001
T1 (*n* = 289)	1	0.71 (0.37–1.37)	0.62 (0.32–1.23)	0.18		0.94 (0.88–1.01)	0.10	
T2 (*n* = 290)	1	1.08 (0.57–2.04)	2.26 (1.22–4.19)	0.011		1.10 (1.03–1.17)	0.006	
T3 (*n* = 289)	1	2.16 (1.11–4.18)	3.00 (1.56–5.81)	0.001		1.14 (1.06–1.21)	< 0.001	
Valine					0.003			0.001
T1 (*n* = 289)	1	0.72 (0.37–1.39)	0.78 (0.40–1.52)	0.48		0.96 (0.90–1.03)	0.25	
T2 (*n* = 290)	1	1.12 (0.59–2.14)	1.88 (1.01–3.50)	0.046		1.09 (1.02–1.16)	0.014	
T3 (*n* = 289)	1	2.10 (1.08–4.08)	2.95 (1.52–5.72)	0.001		1.13 (1.05–1.20)	0.001	

The multivariate logistic regression was used for estimation of ORs and 95% confidence interval (CI)

*BCAAs* branched-chain amino acids, *GRS* genetic risk score, *T2D* type 2 diabetes

^a^Results were adjusted for age, sex, BMI, current drinkers, current smokers, exercise regularly, family history of diabetes, cardiovascular disease, red meat intake, and total energy intake

The results of fasting glucose paralleled to T2D risk. The genetic association with elevated fasting glucose levels increased significantly with higher dietary BCAAs intakes (effect size − 0.07[0.10] mmol/L to 0.39 [0.12] mmol/L per tertile from low to high GRS, *p* for interaction = 0.004) and the GRS was significantly associated with higher fasting glucose in the high and median tertiles of BCAAs intakes but unsignificantly in low tertile (Supplementary Table 4). When GRS was shown in continuous, the changes of fasting glucose per 1 risk allele increment were 0.072 (0.023) mmol/L, 0.068 (0.021) mmol/L, and − 0.019(0.020) mmol/L for individuals with high, medium, and low intakes of total BCAAs, although the trend for interaction was non-significant (Supplementary Table 4). Similar results were observed for individual BCAA.

### Association of dietary BCAAs with T2D risk and fasting glucose according to genetic risk

From another perspective, the association of dietary BCAAs with T2D risk and fasting glucose seemed to be reversed among participants at different genetic risks. For dietary BCAAs in tertiles, the associations between dietary BCAAs and T2D were significantly positive in participants at high genetic risk (total BCAAs: OR 2.90; 95% CI 1.54, 5.47) but changed to negative at low genetic risk (total BCAAs: OR 0.46; 95% CI 0.22, 0.95) (Table 3). When dietary BCAAs were shown in continuous form, the odds ratio of T2D per 10 mg/g protein increment of total BCAAs were 0.95(95% CI: 0.88, 1.03), 1.05(95% CI: 0.98, 1.12), and 1.07(95% CI: 1.00, 1.15) in those with low, moderate and high genetic risk (*p* for interaction = 0.038 for total BCAAs) (Table 3).

**Table 3:** The odds ratio of T2D by dietary BCAAs in classified and continuous form according to GRS in thirds^a^

GRS	Dietary BCAAs in classified mode					Dietary BCAAs in continuous mode		
	T1	T2	T3	*P* for trend	*P* for interaction	Per 10 mg/g protein	*P* value	*P* for interaction
Total BCAAs					0.001			0.038
< 22 (*n* = 293)	1	0.56 (0.28–1.10)	0.46 (0.22–0.95)	0.040		0.95 (0.88–1.03)	0.20	
22–24 (*n* = 279)	1	1.11 (0.59–2.08)	2.22 (1.15–4.31)	0.021		1.05 (0.98–1.12)	0.17	
≥ 25 (*n* = 296)	1	2.19 (1.17–4.10)	2.90 (1.54–5.47)	0.001		1.07 (1.00–1.15)	0.049	
Isoleucine					< 0.001			0.038
< 22 (*n* = 293)	1	0.60 (0.30–1.18)	0.50 (0.24–1.18)	0.07		0.80 (0.57–1.13)	0.21	
22–24 (*n* = 279)	1	1.36 (0.72–2.56)	2.36 (1.21–4.59)	0.012		1.23 (0.92–1.65)	0.16	
≥ 25 (*n* = 296)	1	2.53 (1.34–4.76)	3.29 (1.74–6.25)	< 0.001		1.38 (1.00–1.89)	0.047	
Leucine					0.001			0.039
< 22 (*n* = 293)	1	0.55 (0.28–1.10)	0.47 (0.22–0.98)	0.050		0.91 (0.79–1.05)	0.21	
22–24 (*n* = 279)	1	1.22 (0.65–2.28)	2.24 (1.15–4.35)	0.019		1.09 (0.96–1.24)	0.17	
≥ 25 (*n* = 296)	1	2.23 (1.19–4.19)	3.06 (1.61–5.79)	0.001		1.15 (1.00–1.32)	0.047	
Valine					0.003			0.037
< 22 (*n* = 293)	1	0.59 (0.30–1.17)	0.49 (0.24–1.03)	0.06		0.81 (0.60–1.09)	0.17	
22–24 (*n* = 279)	1	1.12 (0.60–2.10)	2.07 (1.07–4.01)	0.035		1.19 (0.92–1.54)	0.20	
≥ 25 (*n* = 296)	1	1.53 (0.82–2.86)	2.49 (1.33–4.67)	0.004		1.31 (0.99–1.74)	0.06	

The multivariate logistic regression was used for estimation of ORs and 95% confidence interval (CI)

*BCAAs* branched-chain amino acids, *GRS* genetic risk score, *T2D* type 2 diabetes

^a^Results were adjusted for age, sex, BMI, current drinkers, current smokers, exercise regularly, family history of diabetes, cardiovascular disease, red meat intake, and total energy intake

For fasting glucose, the positive association between dietary BCAAs and fasting glucose was strongest in participants with high GRS (0.26 [0.13] mmol/L per tertile, *p* =0.040 for total BCAAs), while the association changed to negative in low GRS (− 0.23[0.10] mmol/L per tertile, *p* = 0.021 for total BCAAs) (Supplementary Table 5). When dietary BCAAs were shown in continuous mode, the changes of fasting glucose per 10 mg/g protein increment of total BCAAs were − 0.040 (0.019), 0.008 (0.020), and 0.038 (0.027) mmol/l for those with low, moderate, and high genetic risk (Supplementary Table 5). Similar results were observed for individual BCAA, but the differences in fasting glucose changes associated with increasing Valine intakes across these subgroups were not evident.

### Genetic association with circulating BCAAs and the interaction between dietary BCAAs and GRS on circulating BCAAs

The GRS was positively associated with the concentrations of circulating total BCAAs and Valine, both in classified and continuous forms (Table 4). The concentrations of circulating total BCAAs and Valine were significantly higher comparing the extreme grades of GRS (*p* for trend = 0.004 and 0.002). The median circulating total BCAAs concentration was 11.8(1.2) in the lowest GRS and 12.0(1.3) in the highest GRS, corresponding 9.9 (1.1) and 10.0 (1.2) for valine. The increments of circulating total BCAAs and valine per grade of GRS were 0.136 (0.047) and 0.145 (0.046). When GRS was in continuous form, changes in plasma total BCAAs and Valine per 1 allele increase in GRS were 0.028 (0.010) and 0.031 (0.009) respectively(*p* for trend = 0.003 and 0.001).

**Table 4:** Circulating BCAAs by classified and continuous mode of GRS^a^

Circulating BCAAs	GRS in classified mode					GRS in continuous mode	
	< 22 (*n* = 293)	22–24 (*n* = 279)	≥ 25 (*n* = 296)	Effect size	*P* for trend	Effect size	*P* value
Total BCAAs	139.5(26.8)	140.5(25.7)	**146.3(31.5)**^b^	**3.6(1.1)**	0.002	**0.028(0.010)**	**0.003**
Isoleucine	24.3(8.6)	23.5(8.76)	24.9 (8.8)	0.4 (0.4)	0.27	0.004(0.007)	0.59
Leucine	16.1(7.1)	15.6(8.32)	15.9(7.9)	0.09(0.3)	0.29	-0.001(0.007)	0.86
Valine	99.1(21.9)	101.4(20.5)	**106.0(25.0)**^b^	**3.0(0.9)**	0.001	**0.031(0.009)**	**0.001**

The general linear model was used for estimation of mean (SD) for circulating BCAAs and linear regression model for β coefficient (SE)

*BCAAs* branched-chain amino acids, *GRS* genetic risk score, *T2D* type 2 diabetes

^a^Results were adjusted for age, sex, BMI, current drinkers, current smokers, exercise regularly, family history of diabetes, cardiovascular disease, red meat intake, and total energy intake.

^b^Comparing to the lowest GRS group, *P* < 0.05.

Furthermore, dietary BCAAs significantly interact with GRS on the level of circulating BCAAs (*p* for interaction = 0.046) (Supplementary Table 6). The association with elevated circulating BCAAs levels increased significantly with higher BCAAs intake (effect size 0.19umol/L to 8.42umol/L per GRS group from the lowest to highest tertile), and the GRS was significantly associated with circulating BCAAs only in the highest tertile of BCAAs intake (*p* for trend < 0.001). In addition, the interaction between dietary and plasma BCAAs on T2D risk was also examined in our study that dietary BCAAs increased T2D risk only when plasma concentrations were also higher, although the interaction was non-significant (Supplementary Table 7).

### Sensitivity analysis

When BCAAs were expressed as absolute intake or the proportion of energy, the interactions between dietary BCAAs and GRS on T2D risk seemed to be unsignificant. From another point of view, in analyses using the BCAAs reported as percentage of total amino acid intake yielded consistent results, the significant interaction remained (*p* for interaction = 0.007) (Supplementary Table 8).

## Discussion

In the current study, we found the significant interaction between dietary BCAAs and genetic predisposition related to T2D risk and fasting glucose in the prospective nested case-control study of Chinese. Our findings show that increasing BCAAs intakes could significantly enhance the genetic association with T2D risk and fasting glucose. Viewed differently, raising BCAAs intakes were associated with increases in T2D risk and fasting glucose, such unfavorable effects were aggravated among participants with high genetic susceptibility while the effect changed to favorable in those with low genetic risk. Furthermore, genetic predisposition to T2D can increase plasma level of BCAAs after adjustment for dietary BCAAs. To our knowledge, this was the first research to examine the interaction between dietary BCAAs and genetic predisposition on T2D and associated factors.

Accordingly, one of the main findings and novelty of our results is that we have found that the genetic association with T2D was influenced by the dietary BCAAs consumed. When BCAAs intakes were heavy, the GRS was significantly associated with higher T2D risk and fasting glucose, while light BCAAs intakes blunted this association. Both observation and intervention studies provide evidence supporting that diet can modify the genetic relationships with T2D and related traits [46, 47]. Instead of considering individual candidate genetic variant in isolation, GRS combines of numerous susceptibility loci of T2D was identified in our study, reflected the polygenic nature of T2D therefore represented a broader picture of genetic susceptibility and was crucial for exploring gene–diet interactions on T2D. Adverse influences of these impairments may be exacerbated under unhealthy dietary habits. Previous studies suggested that high consumption of BCAAs is associated with an increased risk of T2D [20, 21]. A BCAA supplementation in mice was reported to cause insulin resistance, despite a reduced food intake and weight gain [48]. Cell and animal studies found that BCAAs deprivation can improve insulin sensitivity by decreased mTOR/S6K1 and can promote insulin-stimulated glucose transport in muscle and fat and then accelerate the utilization of glucose which may provide potential mechanisms of BCAAs effects [49, 50]. Based on these researches, it is not surprising that in the current study, heavy intakes of BCAAs augmented the change in T2D risk associated with GRS. Moreover, dietary BCAAs significantly interacted with GRS on circulating BCAAs, the risk factors for T2D, and amplified the genetic association with circulating BCAAs which provided further evidence for the reliability of the interaction on T2D risk.

From another point of view, our findings indicated that the effect of higher BCAAs intake on T2D incident was dependent on individual genetic background. In people with a high GRS, increasing BCAAs intakes seemed to exert a harmful effect on T2D and fasting glucose, while the effect changed to protective in those with a low GRS. Previous researches have clearly demonstrated considerable genetic heterogeneity in response to diet and lend support to personalized nutrition according to genotype in the future [51]. Lu Qi et al. found the interaction between genetic predisposition and Western dietary pattern on T2D risk. The Western diet pattern was more strongly correlated to T2D incident among participants in higher GRS but unsignificantly among participants in lower GRS (*p* for interaction = 0.02) [45]. Increasing levels of BCAAs have been enucleated to independently predict future T2D onset [15, 52] and are affected by intakes and catabolism rate meanwhile [19]. BCAAs metabolism primarily occurs in mitochondria of peripheral tissue, thus the function of mitochondrial exerts a notable impact on plasma BCAA level [11]. Branched-chain α-keto acid dehydrogenase (BCKD) is most responsible for catalyzing BCKA decarboxylation, which is the rate-controlling and the first irreversible step in BCAA oxidative catabolism [53, 54]. High BCAA intakes might be beneficial or harmful for metabolic health, depending on the mitochondrial metabolism capacity, since individuals with complete metabolism could tolerate heavy BCAA intake well because of the reserve function of BCKDC and that under normal status, BCKD can be activated by excess substrate [55]. Insulin is the prominent regulator of BCAAs metabolism by influencing the rate of appearance and clearance together with a decreased activity of mitochondrial catabolic enzymes [19]. Yuvaraj et al. used a GRS as an instrumental variable for insulin resistance and found that increased insulin resistance drives higher circulating BCAA levels [29]. Research about a cluster of BCAA metabolites also suggested a primary alteration of BCAA metabolism in insulin resistance [14]. Therefore, mitochondrial dysfunction in those with or at T2D risk will reduce the capacity of mitochondria to catabolize BCAAs, leading to the BCAAs levels raising in blood [15, 18], supporting our result that the higher GRS was associated with higher circulating BCAAs levels. Under this condition, heavy BCAAs intakes appear particularly detrimental as they increase the substrate load for mitochondrial oxidation then results in mitochondrion dysfunction as well as impaired insulin secretion and action [56]. But for those with contact metabolism of BCAAs, a previous study showed that dietary BCAAs may be favorable by improving muscle protein synthesis [56] as well as glucose metabolism [23], as the higher BCAAs intakes associated with protection from T2D among those with low GRS in our study. Moreover, our results showed that high dietary BCAAs were significantly related to an increased risk of T2D when plasma concentrations were also higher although the trend for interaction was unsignificant. This result further corroborated the report by Zhang et al. that for women with a history of gestational diabetes, high dietary BCAA intake was positively associated with T2D incident in those accompanying with high circulating levels but not in those with low circulating levels [57].

In our study, we expressed dietary BCAAs as a percentage of total protein intakes rather than a percentage of total energy or absolute intakes. Firstly, dietary BCAA comes from protein, so they correlated with protein by their nature and the correlation with protein (*r* = 0.99) was stronger than with energy (*r* = 0.86). Therefore, Nutrient density can help to reduce the likelihood of multicollinearity and provide a better understanding of the effect of BCAAs which is preferable than absolute intakes [58]. Secondly, protein consists of multiple amino acids which may be collinear by their nature. Previous studies found that BCAAs can interplay with other amino acids in a direct or indirect manner, such as the direct reciprocity with glycine metabolism or competition with lysine in the transport system in the intestine [59, 60]. So the relative intake of BCAAs adjusting for total protein intake can remove the mask from protein and other amino acids to some extent and preserve a meaningful impact for BCAA [61]. Thirdly, although BCAAs were abundant in a variety of protein sources, confirmation of the need for particularly high intakes of BCAAs at each meal, particularly within a calorie-restricted diet, could have implications for choosing a protein source. Since the content of BCAAs in isolate whey protein and milk protein are much higher than in wheat protein (26%, 21% vs 15% of protein), larger proportion of whey protein or milk protein is recommended within a calorie-restricted diet [62]. Finally, when we replaced the proportions of total protein with proportions of total amino acids, the interaction remained, which demonstrated the robustness of our findings. On these bases, expressing BCAAs as proportions of total protein may be a more useful method of evaluation, because they address multicollinearity of nutrients, consider about the complexity of human diet, and can provide a useful target to interventions for diabetes prevention [63].

The SNPs used to build GRS in our research came from candidate gene studies and GWAS studies. First, there were few GWAS studies focusing on Chinese, and most of them were undertaking in individuals of European ancestry. Like Khera et al. proposed that allele frequencies, linkage disequilibrium patterns, and effect sizes of common polymorphisms varied with ancestry [64], using the SNPs entirely coming from existing GWAS studies may not well suitable for our research. Secondly, most SNPs we choosing from candidate gene studies have been illustrated about the physiological mechanism of T2D risk and proved to be a reliable risk factor. However, the diabetes-related SNPs finding from GWAS studies may show nominal association and frequently be ignored of their biological functions. Finally, numerous researches used SNPs from candidate gene studies and GWAS studies to predict disease risk, which may show the rationality of this method [65–67]. Furthermore, comparing participants in the lowest tertile of GRS we built, the odds ratio of the highest tertile was 1.72 (95% CI 1.16–2.56) in our research, which may show the GRS can credibly represent T2D hereditary susceptibility.

Strengths in our study include the prospective nested case-control design, which can identify the exposures before T2D diagnosis and then alleviate the effect of disease and related treatments on participants' diet and metabolic profiles. Several limitations need to be addressed. First, the precise mechanisms for the observed interactions remain unclear. Although the biological mechanisms mentioned above could be plausible that may contribute to demonstrating the observed gene-diet interaction, we believe that it is not full and that further research has to be focused on this point. Secondly, statistically significant results should be accepted with caution as in our study, we did not examine the mitochondrial function to test the hypothesis. Furthermore, our study population was all from north China which may limit the generalization of our findings to other populations, since allele frequencies, linkage disequilibrium patterns, and effect sizes of SNPs varying with populations. So replications of the interaction in other populations are needed in the future.

## Conclusion

Our study firstly provides evidence from a prospective nested case-control study of China that dietary BCAAs could magnify the genetic association with T2D risk and fasting glucose, and the adverse effects of high BCAAs intakes on T2D and fasting glucose were exacerbated in people with high genetic susceptibility, but change to favorable in those with low genetic susceptibility. Our findings emphasize the importance of reducing BCAAs intakes in people with great genetic susceptibility but modestly increasing BCAAs in those with low genetic susceptibility to prevent T2D more effectively. Further studies are needed to apply these findings widely in public health practice.

## Supplementary Information

**Additional file 1: Supplementary Table 1.** The content of individual BCAA and protein in different food. **Supplementary Table 2.** Characteristics of genetic variants associated with type 2 diabetes. **Supplementary Table 3.** Tertiles of GRS and dietary BCAAs in relation to T2D risk and fasting glucose. **Supplementary Table 4**. The association of GRS in tertiles and in continuous with fasting glucose according to BCAAs intakes in thirds. **Supplementary Table 5.** The association of dietary BCAAs in tertiles and in continuous with fasting glucose according to GRS in thirds. **Supplementary Table 6.** The interaction between dietary BCAAs and GRS on circulating BCAAs level. **Supplementary Table 7.** The odds ratio of T2D according to categories of circulating BCAAs and dietary BCAAs in thirds. **Supplementary Table 8.** Interaction between dietary BCAAs expressed in three other ways and GRS on T2D risk.

## Data Availability

All of the data are available with a reasonable request from the corresponding author

## Abbreviations

BCAAs: Branched-chain amino acids
BCKD: Branched-chain α-keto acid dehydrogenase
GRS: Genetic risk score
GWAS: Genome-wide association study
OGTT: Oral glucose tolerance test
SNP: Single nucleotide polymorphism
T2D: Type 2 diabetes
